# Diagnostic Performance of BD Phoenix CPO Detect Panels for Detection and Classification of Carbapenemase-Producing Gram-Negative Bacteria

**DOI:** 10.1128/spectrum.00897-23

**Published:** 2023-05-10

**Authors:** Mika Murata, Kosuke Kosai, Norihiko Akamatsu, Yumiko Matsuyama, Mitsuharu Oda, Atsushi Wakamatsu, Koichi Izumikawa, Hiroshi Mukae, Katsunori Yanagihara

**Affiliations:** a Department of Laboratory Medicine, Nagasaki University Hospital, Nagasaki, Japan; b Nippon Becton, Dickinson Company, Ltd., Minato, Tokyo, Japan; c Department of Infectious Diseases, Nagasaki University Graduate School of Biomedical Sciences, Nagasaki, Japan; d Department of Respiratory Medicine, Nagasaki University Graduate School of Biomedical Sciences, Nagasaki, Japan; Weill Cornell Medicine

**Keywords:** carbapenem resistance, carbapenemase, Ambler classification, automated system, rapid detection

## Abstract

BD Phoenix CPO Detect panels can identify and classify carbapenemase-producing organisms (CPOs) simultaneously with antimicrobial susceptibility testing (AST) for Gram-negative bacteria. Detection and classification of carbapenemase producers were performed using the BD Phoenix CPO Detect panels NMIC/ID-441 for *Enterobacterales*, NMIC/ID-442 for nonfermenting bacteria, and NMIC-440 for both. The results were compared with those obtained using comparator methods. A total of 133 strains (32 Klebsiella pneumoniae, 37 Enterobacter cloacae complex, 33 Pseudomonas aeruginosa, and 31 Acinetobacter baumannii complex strains), including 60 carbapenemase producers (54 imipenemases [IMPs] and 6 OXA type), were analyzed. Using panels NMIC-440 and NMIC/ID-441 or NMIC/ID-442, all 54 IMP producers were accurately identified as CPOs (positive percent agreement [PPA], 100.0%; 54/54). Among the 54 IMP producers identified as CPOs using panels NMIC-440 and NMIC/ID-441, 12 and 14 *Enterobacterales* were not resistant to carbapenem, respectively. Among all 54 IMP producers, 48 (88.9%; 48/54) were correctly classified as Ambler class B using panel NMIC-440. Using panels NMIC-440 and NMIC/ID-442, all four OXA-23-like carbapenemase-producing A. baumannii complex strains (100.0%, 4/4) were correctly identified as CPOs, and three (75.0%, 3/4) were precisely classified as class D using panel NMIC-440. Both carbapenemase producers harboring the *bla*_ISAba1-OXA-51-like_ gene were incorrectly identified as non-CPOs using panels NMIC-440 and NMIC/ID-442. For detecting carbapenemase producers, the overall PPA and negative percent agreement (NPA) between panel NMIC-440 and the comparator methods were 96.7% (58/60) and 71.2% (52/73), respectively, and the PPA and NPA between panels NMIC/ID-441 or NMIC/ID-442 and the comparator methods were 96.7% (58/60) and 74.0% (54/73), respectively. BD Phoenix CPO Detect panels can successfully screen carbapenemase producers, particularly IMP producers, regardless of the presence of carbapenem resistance and can be beneficial in routine AST workflows.

**IMPORTANCE** Simple and efficient screening methods of detecting carbapenemase producers are required. BD Phoenix CPO Detect panels effectively screened carbapenemase producers, particularly IMP producers, with a high overall PPA. As the panels enable automatic screening for carbapenemase producers simultaneously with AST, the workflow from AST to confirmatory testing for carbapenemase production can be shortened. In addition, because carbapenem resistance varies among carbapenemase producers, the BD Phoenix CPO Detect panels, which can screen carbapenemase producers regardless of carbapenem susceptibility, can contribute to the accurate detection of carbapenemase producers. Our results report that these panels can help streamline the AST workflow before confirmatory testing for carbapenemase production in routine microbiological tests.

## INTRODUCTION

The spread of carbapenemase-producing Gram-negative bacteria poses a significant global threat. Carbapenems, which are broad-spectrum, highly reliable antimicrobials for treating serious infections, are degraded by carbapenemases, impairing their efficacy. There are several types of carbapenemases, such as Klebsiella pneumoniae carbapenemases (KPCs), metallo-β-lactamases (MBLs) (including imipenemase [IMP], Verona integron-encoded MBL [VIM], and New Delhi MBL [NDM]), and OXA-type carbapenemases (1). In Japan, IMP is a major carbapenemase type produced by K. pneumoniae, Enterobacter cloacae complex, and Pseudomonas aeruginosa strains (2–5). In addition to IMP, several OXA-type carbapenemases have been identified in Acinetobacter species (6, 7).

The detection of carbapenemase producers is crucial for appropriate antimicrobial therapy and infection control. However, it is difficult to identify them using antimicrobial susceptibility testing (AST) alone because carbapenemase producers may show various levels of MICs for carbapenems, depending on the gene expression, efficacy of carbapenem hydrolysis, and presence of other resistance mechanisms, such as permeability (1, 8). We previously reported that carbapenemase-producing K. pneumoniae and E. cloacae complex strains could be overlooked if screened based on carbapenem resistance detected using the broth microdilution method (9). Therefore, simple and efficient screening methods that are independent of the MIC results are required in daily practice.

In this study, we evaluated BD Phoenix CPO Detect panels, which enable the detection and classification of carbapenemase-producing organisms (CPOs) simultaneously with AST for Gram-negative bacteria, and discuss their usefulness in a routine microbiological testing workflow.

## RESULTS

A total of 133 (32 K. pneumoniae, 37 E. cloacae complex, 33 P. aeruginosa, and 31 Acinetobacter baumannii complex) strains were included in this study, among which, 60 strains (15, 20, 14, and 11, respectively) were identified as true carbapenemase producers using the comparator methods. All carbapenemases observed in K. pneumoniae, E. cloacae complex, and P. aeruginosa strains belonged to group IMP-1, including the IMP-6 observed in only one P. aeruginosa strain. Five *bla*_IMP-1_ group, four *bla*_OXA-23-like_, and two *bla*_ISAba1-OXA-51-like_ genes were included in the 11 carbapenemase-producing A. baumannii complex strains. Tables 1 to 3 show the results of comparisons between panel NMIC-440 and the comparator methods for detecting carbapenemase producers. Using panel NMIC-440, all 54 true IMP producers (15 K. pneumoniae, 20 E. cloacae complex, 14 P. aeruginosa, and five A. baumannii complex strains) were identified as CPOs. Among these 54, 42 and 12 were carbapenem-resistant and nonresistant strains, respectively. Furthermore, using panel NMIC-440, 48 (88.9%, 48/54) were correctly classified as class B, and 6 (11.1%, 6/54) were unclassified. In the A. baumannii complex strains not harboring the *bla*_IMP-1_ group, four true OXA-23-like carbapenemase producers were identified as CPOs using panel NMIC-440; among these, three were classified as class D, and one was incorrectly classified as class B. Using panel NMIC-440, two true carbapenemase producers harboring the *bla*_ISAba1-OXA-51-like_ gene were not identified as CPOs. Meanwhile, among the 73 true non-carbapenemase producers, panel NMIC-440 incorrectly detected 21 (5 K. pneumoniae, 7 P. aeruginosa, and 9 A. baumannii complex) strains as CPOs, among which 18 were incorrectly classified as belonging to several classes (class A, 5 P. aeruginosa strains; class B, 3 K. pneumoniae strains and 1 P. aeruginosa strain; class D, 9 A. baumannii complex strains), whereas three remained unclassified. With respect to carbapenem resistance, among the 58 true carbapenemase producers with correct detection using the panel, 12 (2 K. pneumoniae and 10 E. cloacae complex strains) were not resistant to carbapenem. Conversely, among the 52 true non-carbapenemase producers correctly determined as non-CPOs using the panel, four (two E. cloacae complex and two P. aeruginosa) strains exhibited carbapenem resistance.

**TABLE 1:** Detection and classification of CPOs for true carbapenemase producers using panel NMIC-440^*a*^

Species	Comparator method results		NMIC-440 results			No. of strains
	Type	Class	CPO	Class	Carbapenem susceptibility	
K. pneumoniae	IMP	B	Pos	B	R	10
	IMP	B	Pos	B	NR	2
	IMP	B	Pos	UC	R	3
E. cloacae complex	IMP	B	Pos	B	R	10
	IMP	B	Pos	B	NR	10
P. aeruginosa	IMP	B	Pos	B	R	11
	IMP	B	Pos	UC	R	3
A. baumannii complex	IMP	B	Pos	B	R	5
	OXA-23-like	D	Pos	B	R	1
	OXA-23-like	D	Pos	D	R	3
	ISAba1-OXA-51-like	D	Neg		R	1
	ISAba1-OXA-51-like	D	Neg		NR	1

a CPO, carbapenemase-producing organism; UC, unclassified; R, resistant; NR, not resistant.

**TABLE 2:** Detection and classification of CPOs for true non-carbapenemase producers using panel NMIC-440^*a*^

Species	NMIC-440 results			No. of strains
	CPO	Class	Carbapenem susceptibility	
K. pneumoniae	Pos	B	NR	3
	Pos	UC	R	1
	Pos	UC	NR	1
	Neg		NR	12
E. cloacae complex	Neg		R	2
	Neg		NR	15
P. aeruginosa	Pos	A	R	5
	Pos	B	R	1
	Pos	UC	R	1
	Neg		R	2
	Neg		NR	10
A. baumannii complex	Pos	D	R	9
	Neg		NR	11

a CPO, carbapenemase-producing organism; UC, unclassified; R, resistant; NR, not resistant.

**TABLE 3:** Detection and classification summary of CPOs using panels NMIC-440, NMIC/ID-441, or NMIC/ID-442^*a*^

Comparator method results		No. of strains	NMIC-440 results					NMIC/ID-441 or NMIC/ID-442 results^*b*^
Type	Class		No. of CPOs	No. of strains in class:				No. of CPOs
				A	B	D	UC	
IMP	B	54	54	0	48	0	6	54
OXA-23-like	D	4	4	0	1	3	0	4
ISAba1-OXA-51-like	D	2	0	0	0	0	0	0
Nonproducer		73	21	5	4	9	3	19

a CPOs, carbapenemase-producing organisms; UC, unclassified.

b NMIC/ID-441 or -442 was used for *Enterobacterales* or nonfermenting bacteria, respectively.

The performance of panel NMIC-440 for detecting carbapenemase producers in each bacterial group is presented in Table 4. The positive percent agreements (PPAs) between panel NMIC-440 and the comparator methods were 100.0% (15/15, 20/20, and 14/14, respectively) for K. pneumoniae, E. cloacae complex, and P. aeruginosa strains, and 81.8% (9/11) for A. baumannii complex strains. Conversely, the negative percent agreement (NPA) between the two varied from 55.0% (11/20) in A. baumannii complex strains to 100.0% (17/17) in E. cloacae complex strains. The overall PPA and NPA were 96.7% (58/60) and 71.2% (52/73), respectively.

**TABLE 4:** Diagnostic performance of panel NMIC-440 for the detection of carbapenemase producers^*a*^

Species	No. of carbapenemase producers^*b*^		No. of nonproducers^*b*^		PPA (95% CI)	NPA (95% CI)
	CPO^*c*^	Non-CPO^*c*^	CPO^*c*^	Non-CPO^*c*^		
K. pneumoniae	15	0	5	12	100.0 (78.2–100.0)	70.6 (44.0–89.7)
E. cloacae complex	20	0	0	17	100.0 (83.2–100.0)	100.0 (80.5–100.0)
P. aeruginosa	14	0	7	12	100.0 (76.8–100.0)	63.2 (38.4–83.7)
A. baumannii complex	9	2	9	11	81.8 (48.2–97.7)	55.0 (31.5–76.9)
Total	58	2	21	52	96.7 (88.5–99.6)	71.2 (59.4–81.2)

a CPO, carbapenemase-producing organism; PPA, positive percent agreement; NPA, negative percent agreement; CI, confidence interval.

b Determined using comparator methods.

c Determined using panel NMIC-440.

The detection results of carbapenemase producers using panels NMIC/ID-441 or NMIC/ID-442 and the comparator methods are shown in Tables 3, 5, and 6. The results using panel NMIC/ID-441 for K. pneumoniae and E. cloacae complex strains were fully concordant with those using panel NMIC-440. Using panel NMIC/ID-442, all 19 true IMP producers (14 P. aeruginosa and 5 A. baumannii complex strains) were identified as CPOs. Four true OXA-23-like carbapenemase-producing A. baumannii complex strains were accurately detected as CPOs. Both true carbapenemase producers harboring the *bla*_ISAba1-OXA-51-like_ gene were incorrectly identified as non-CPOs using panel NMIC/ID-442. In contrast, 14 true non-carbapenemase producers (five P. aeruginosa and nine A. baumannii complex strains) were incorrectly identified as CPOs using panel NMIC/ID-442. Among the 58 carbapenemase producers that were detected successfully using panels NMIC/ID-441 or NMIC/ID-442, 14 strains (two K. pneumoniae and 12 E. cloacae complex strains) were carbapenem nonresistant. Among the 54 non-carbapenemase producers with correct determination using panels NMIC/ID-441 or NMIC/ID-442, six (two E. cloacae complex, three P. aeruginosa, and one A. baumannii complex) strains showed carbapenem resistance.

**TABLE 5:** Detection of CPOs for true carbapenemase producers using panels NMIC/ID-441 or -442^*a*^

Species	Comparator method results		NMIC/ID-441 or NMIC/ID-442 results^*b*^		No. of strains
	Type	Class	CPO	Carbapenem susceptibility	
K. pneumoniae	IMP	B	Pos	R	13
	IMP	B	Pos	NR	2
E. cloacae complex	IMP	B	Pos	R	8
	IMP	B	Pos	NR	12
P. aeruginosa	IMP	B	Pos	R	14
A. baumannii complex	IMP	B	Pos	R	5
	OXA-23-like	D	Pos	R	4
	ISAba1-OXA-51-like	D	Neg	R	1
	ISAba1-OXA-51-like	D	Neg	NR	1

a CPO, carbapenemase-producing organism; R, resistant; NR, not resistant.

b Determined using panel NMIC/ID-441 for *Enterobacterales* strains or NMIC/ID-442 for nonfermenting bacteria.

**TABLE 6:** Detection of CPOs for true non-carbapenemase producers using panels NMIC/ID-441 or -442^*a*^

Species	NMIC/ID-441 or NMIC/ID-442 results^*b*^		No. of strains
	CPO	Carbapenem susceptibility	
K. pneumoniae	Pos	R	2
	Pos	NR	3
	Neg	NR	12
E. cloacae complex	Neg	R	2
	Neg	NR	15
P. aeruginosa	Pos	R	5
	Neg	R	3
	Neg	NR	11
A. baumannii complex	Pos	R	8
	Pos	NR	1
	Neg	R	1
	Neg	NR	10

a CPO, carbapenemase-producing organism; R, resistant; NR, not resistant.

b Determined using panel NMIC/ID-441 for *Enterobacterales* strains or NMIC/ID-442 for nonfermenting bacteria.

The concordance in the detection of carbapenemase producers between panels NMIC/ID-441 or NMIC/ID-442 and the comparator methods is presented in Table 7. Based on the full concordance in the detection of carbapenemase producers in K. pneumoniae and E. cloacae complex strains between panels NMIC-440 and NMIC/ID-441, the performance was the same between these two panels (Tables 4 and 7). The detection results of panels NMIC-440 and NMIC/ID-442 for carbapenemase producers in nonfermenting bacteria were different only for the two P. aeruginosa and two A. baumannii complex strains. Consequently, the performance was similar between the two panels in the PPA (100.0%; 14/14) for the P. aeruginosa strains and in the PPA (81.8%; 9/11) and NPA (55.0%; 11/20) for the A. baumannii complex strains. The NPA between panel NMIC/ID-442 and the comparator methods was 73.7% (14/19) for P. aeruginosa.

**TABLE 7:** Diagnostic performance of panels NMIC/ID-441 and -442 for the detection of carbapenem producers^*a*^

Species	No. of carbapenemase producers^*b*^		No. of nonproducers^*b*^		PPA (95% CI)	NPA (95% CI)
	CPO^*c*^	Non-CPO^*c*^	CPO^*c*^	Non-CPO^*c*^		
K. pneumoniae	15	0	5	12	100.0 (78.2–100.0)	70.6 (44.0–89.7)
E. cloacae complex	20	0	0	17	100.0 (83.2–100.0)	100.0 (80.5–100.0)
P. aeruginosa	14	0	5	14	100.0 (76.8–100.0)	73.7 (48.8–90.9)
A. baumannii complex	9	2	9	11	81.8 (48.2–97.7)	55.0 (31.5–76.9)
Total	58	2	19	54	96.7 (88.5–99.6)	74.0 (62.4–83.5)

a CPO, carbapenemase-producing organism; PPA, positive percent agreement; NPA, negative percent agreement; CI, confidence interval.

b Determined using comparator methods.

c Determined using panel NMIC/ID-441 for *Enterobacterales* strains or NMIC/ID-442 for nonfermenting bacteria.

The overall PPA and NPA between panels NMIC/ID-441 or NMIC/ID-442 and the comparator methods were 96.7% (58/60) and 74.0% (54/73), respectively.

## DISCUSSION

This study demonstrated the performance of BD Phoenix CPO Detect panels, NMIC/ID-441 (*Enterobacterales*), NMIC/ID-442 (nonfermenting bacteria), and NMIC-440 (both), in detecting carbapenemase producers. Our results revealed a high overall PPA (96.7%; 58/60) for detecting carbapenemase producers between the panels and the comparator methods (Tables 4 and 7). In this study, the main carbapenemase type was IMP, owing to regional epidemiology, and using panels NMIC-440 and NMIC/ID-441 or NMIC/ID-442, all 54 IMP producers were accurately identified as CPOs (PPA, 100.0%; 54/54) (Tables 1 to 3, 5, and 6). These results are supported by previous reports that BD Phoenix CPO Detect panels correctly identified all IMP producers examined as CPOs (10, 11) and indicate that all panels evaluated in this study (NMIC-440, NMIC/ID-441, and NMIC/ID-442) are useful in screening IMP-producing K. pneumoniae, E. cloacae complex, P. aeruginosa, and A. baumannii complex strains. In addition, among the 54 IMP producers identified as CPOs using panels NMIC-440 and NMIC/ID-441 or NMIC/ID-442, 12 and 14 *Enterobacterales* strains were not resistant to carbapenem, respectively, indicating that the detection of carbapenemase producers was successful regardless of the presence of carbapenem resistance. Furthermore, among the 54 IMP producers, 48 (88.9%; 48/54) were accurately classified as class B using panel NMIC-440 (Tables 1 to 3, 5, and 6), which is supported by previous reports that BD Phoenix CPO Detect panels successfully classified 38.1% to 93.3% of IMP producers as class B (10, 11). In this study, using panels NMIC-440 and NMIC/ID-442, all four OXA-23-like carbapenemase-producing A. baumannii complex strains (100.0%; 4/4) were identified as CPOs, and three (75.0%; 3/4) were successfully classified as class D using panel NMIC-440. Previous studies have described the accurate identification of 95.5% to 100.0% of A. baumannii complex strains with OXA-23 as CPOs using BD Phoenix CPO Detect panels and classification of 63.6% to 75.0% as class D (10, 11), which is consistent with our results.

Moreover, our results show that the overall NPAs between the panels and comparator methods were 71.2% (52/73) and 74.0% (54/73) (Tables 4 and 7). Similarly, previous studies reported that BD Phoenix CPO Detect panels accurately identified 68.6% to 99.5% of true non-carbapenemase producers as non-CPOs (10–12). In this study, among the 21, 5, and 14 false positives for detecting CPOs obtained using panels NMIC-440, NMIC/ID-441, and NMIC/ID-442, 17 (81.0%; 17/21), 2 (40.0%; 2/5), and 13 (92.9%; 13/14) strains showed carbapenem resistance, respectively.

Collectively, based on their detection performance with high PPAs compared to the comparator methods, panels NMIC-440, NMIC/ID-441, and NMIC/ID-442 can be used to detect CPOs, particularly IMP producers, without omission. Meanwhile, we should report the results to clinicians with regard to carbapenemase production and classification for strains detected as CPOs using these panels after performing confirmatory tests, in consideration of NPAs. However, confirmatory tests are usually performed after determining antimicrobial resistance using AST and require additional procedures and time. Therefore, rapid and highly sensitive screening methods are desirable to determine whether additional confirmatory tests are required. In this regard, the BD Phoenix CPO Detect panels, which screen IMP producers simultaneously with AST, with high PPAs in comparison with comparator methods, might be beneficial in the daily AST workflow. In addition, carbapenemase producers may be overlooked if confirmatory tests are performed based on AST results, because not all carbapenemase producers exhibit carbapenem resistance. Therefore, BD Phoenix CPO Detect panels, which can screen for carbapenemase producers regardless of carbapenem susceptibility, can contribute to their detection without omission, thereby preventing their proliferation in medical settings. In particular, the panels might be helpful for regions with high IMP prevalence based on the findings of this study.

There are a few limitations of this study. Because we mainly analyzed IMP producers as carbapenemase producers in this study, we could not fully evaluate other types of carbapenemases. Additionally, we used a modified carbapenem inactivation method (mCIM) as a comparator method for detecting carbapenemase-producing A. baumannii complex strains; however, other methods might be appropriate in some cases. Finally, if IMP is not the dominant carbapenemase, the performance data reported here, such as the PPA and NPA, might change.

In conclusion, we evaluated the performance of BD Phoenix CPO Detect panels (NMIC-440, NMIC/ID-441, and NMIC/ID-442) for the detection and classification of carbapenemase producers. These panels were successfully used to screen for carbapenemase producers, particularly IMP producers, and could be beneficial in routine AST workflows.

## MATERIALS AND METHODS

### Bacterial strains and comparator methods.

We used bacterial strains isolated between 2009 and 2017 at Nagasaki University Hospital and stored at −80°C. Bacteria were cultured on Nissui separated plate sheep blood agar/chocolate agar EXII (Nissui Pharmaceutical Co., Ltd.) in 5% CO_2_ at 35 ± 2°C for 18 to 24 h. None of the groups contained duplicate isolates from a single patient. Carbapenemase genes were examined using a multiplex PCR assay kit, the Cica Geneus carbapenemase genotype detection kit 2 (Kanto Chemical Co., Inc.), which can detect *bla*_IMP-1_ group, *bla*_IMP-6_, *bla*_VIM_ group, *bla*_NDM_ group, *bla*_OXA-48_ group, *bla*_KPC_ group, and *bla*_GES_ group carbapenemases, according to the manufacturer’s instructions. Amplification was performed under the following conditions: 1 min at 94°C; 30 cycles of 15 s at 94°C, 15 s at 63°C, and 40 s at 72°C. In addition, *bla*_OXA-23-like_ and *bla*_ISAba1-OXA-51-like_ genes were examined using PCR, and the following primers were used (13, 14): OXA-23-like forward, 5′-GATCGGATTGGAGAACCAGA-3′; OXA-23-like reverse, 5′-ATTTCTGACCGCATTTCCAT-3′; ISAba1 forward, 5′-CACGAATGCAGAAGTTG-3′; and OXA-51-like reverse, 5′-TGGATTGCACTTCATCTTGG-3′. Amplification was conducted under the following conditions: 5 min at 94°C; 30 cycles of 25 s at 94°C, 40 s at 52°C or 58°C, and 50 s at 72°C; and 6 min at 72°C for the final extension.

Carbapenemase production was examined using a modified carbapenem inactivation method (mCIM), according to Clinical and Laboratory Standards Institute (CLSI) guideline M100-ED32, which indicates that the method can be used for *Enterobacterales* and P. aeruginosa. To determine carbapenemase production in A. baumannii complex strains, we preliminarily compared the performance of mCIM and CIMTris, which is another modified CIM (15), for identifying A. baumannii complex strains carrying carbapenemase genes as carbapenemase producers. Among the 16 A. baumannii complex strains carrying *bla*_IMP-1_ group, *bla*_OXA-like_, or *bla*_ISAba1-OXA-51-like_ carbapenemase genes (5, 4, and 7 isolates, respectively), 11 (68.8%; 11/16) and 7 (43.8%; 7/16) tested positive using mCIM and CIMTris, respectively. Using both methods, all 20 A. baumannii strains not carrying those genes were determined as negative. Because mCIM was found to be more sensitive, we used mCIM to detect carbapenemase production in A. baumannii complex strains in this study. True carbapenemase producers were defined as strains that tested positive for both carbapenemase genes and mCIM, whereas true non-carbapenemase producers were defined as strains that tested negative for both carbapenemase genes and mCIM. We excluded strains for which the results were not concordant between PCR and mCIM.

### Assay using BD Phoenix CPO Detect panels.

BD Phoenix CPO Detect panels NMIC-440, NMIC/ID-441, and NMIC/ID-442 detect CPOs simultaneously with AST for Gram-negative bacteria. In addition, panel NMIC-440 concurrently provides Ambler classification results upon detecting a carbapenemase producer. We used panel NMIC/ID-441 for *Enterobacterales* strains, NMIC/ID-442 for nonfermenting bacteria, and NMIC-440 for both.

Assays using the panels were performed according to the manufacturer’s instructions. Briefly, colonies were suspended in a BD Phoenix ID broth, and the suspension was adjusted to a McFarland standard of approximately 0.25. After the BD Phoenix AST indicator solution was added to the BD Phoenix AST broth, 50 μL of the prepared bacterial suspension was transferred to the broth. After the mixture was injected, the panels were loaded into the BD Phoenix M50 instrument, which is an automated bacterial identification and AST system. The system automatically performs assays, determines the results of MICs, detects CPOs, and provides Ambler classification results based on the specifications of each panel.

Resistance to imipenem and meropenem was determined according to CLSI definitions (MICs of ≥4 μg/mL for K. pneumoniae and E. cloacae complex strains; MICs of ≥8 μg/mL for P. aeruginosa and A. baumannii complex strains). Carbapenem-resistant strains were defined as strains exhibiting resistance to either imipenem or meropenem. Susceptible and intermediate strains were classified as not resistant in this study.

### Comparisons between BD Phoenix CPO Detect panels and the comparator methods.

The results of BD Phoenix CPO Detect panels for carbapenemase producers were compared to those of the comparator methods, and positive and negative percent agreements (PPA and NPA) were calculated as follows: PPA, the number of concordant positive results in the detection of carbapenemase producers divided by the number of all positive results in it obtained using comparator methods; NPA, the number of concordant negative results in the detection of carbapenemase producers divided by the number of all negative results in it obtained using comparator methods (16). The 95% confidence intervals were calculated using R version 4.2.2 (17).
